# Gene-Boosted Assembly of a Novel Bacterial Genome from Very Short Reads

**DOI:** 10.1371/journal.pcbi.1000186

**Published:** 2008-09-26

**Authors:** Steven L. Salzberg, Daniel D. Sommer, Daniela Puiu, Vincent T. Lee

**Affiliations:** 1 Center for Bioinformatics and Computational Biology, University of Maryland, College Park, Maryland, United States of America; 2 Department of Cell Biology and Molecular Genetics, University of Maryland, College Park, Maryland, United States of America; University of Washington, United States of America

## Abstract

Recent improvements in technology have made DNA sequencing dramatically faster and more efficient than ever before. The new technologies produce highly accurate sequences, but one drawback is that the most efficient technology produces the shortest read lengths. Short-read sequencing has been applied successfully to resequence the human genome and those of other species but not to whole-genome sequencing of novel organisms. Here we describe the sequencing and assembly of a novel clinical isolate of *Pseudomonas aeruginosa*, strain PAb1, using very short read technology. From 8,627,900 reads, each 33 nucleotides in length, we assembled the genome into one scaffold of 76 ordered contiguous sequences containing 6,290,005 nucleotides, including one contig spanning 512,638 nucleotides, plus an additional 436 unordered contigs containing 416,897 nucleotides. Our method includes a novel gene-boosting algorithm that uses amino acid sequences from predicted proteins to build a better assembly. This study demonstrates the feasibility of very short read sequencing for the sequencing of bacterial genomes, particularly those for which a related species has been sequenced previously, and expands the potential application of this new technology to most known prokaryotic species.

## Author Summary

In this paper we demonstrate that a bacterial genome, *Pseudomonas aeruginosa*, can be decoded using very short DNA sequences, namely, those produced by the newest generation of DNA sequencers such as the Solexa sequencer from Illumina. Our method includes a novel algorithm that uses the protein sequences from other species to assist the assembly of the new genome. This algorithm breaks up the genome into gene-sized chunks that can be put back together relatively easily, even from sequence fragments as short as 30 bases of DNA. We also take advantage of the genomes of related species, using them as reference strains to assist the assembly. By combining these and other techniques, we were able to assemble 94% of the 6.7 million bases of *P. aeruginosa* into just 76 large pieces. The remaining 6% is contained in 436 smaller fragments. We have made all of our software available for free under open-source licenses, and we have deposited the newly assembled genome in the public GenBank database.

## Introduction

Genome sequencing technology has moved into a new era with the introduction of extremely fast sequencing technologies that can produce over one billion base pairs (bp) of DNA in a single run. Some of the fastest methods today, based on strategies such as cyclic reversible termination [1] and ligation-based sequencing [2], produce the shortest read lengths, ranging from 15–50 bp. These lengths are sufficient for resequencing projects, including efforts to sample the human population, but they have yet to prove as useful for sequencing of novel species. The difficulty is that no existing assembly algorithms can accurately reconstruct a genome from such short reads [3].

The first published report of a bacterial genome sequence from “short” reads used pyrosequencing technology, which was able to generate reads averaging 110 bp. That study [4] demonstrated the feasibility of assembling the small bacterial genome of *Mycoplasma genitalium* (580,069 bp) from reads that covered the genome 40-fold. This combination of coverage and read length allowed Margulies et al. to generate contiguous stretchs of DNA (contigs) averaging 22.4 kilobases (kb). Results using pyrosequencing have improved steadily as read lengths have increased to 250 bp and longer, but the difficulty of *de novo* assembly has raised questions about the utility of alternative sequencing technologies—those that produce reads shorter than 50 bp—for genome sequencing projects.

Assembly of novel strains and species—where the genome has not previously been sequenced—from very short reads has proven more difficult, although simulation studies have indicated that it should be possible [5]. A recent study showed that a combination of pyrosequencing reads (average length 102 bp) and paired-end sequencing could be used to assemble a 4 million base pair (Mbp) genome into just 139 contigs, linked together in 22 scaffolds [6]. Another recent effort used a hybrid strategy that mixed pyrosequencing (110 bp reads) and traditional Sanger sequencing to produce draft assemblies of marine microbes [7]. In contrast, the very short reads generated by the Solexa Sequence Analyzer have thus far been useful primarily for polymorphism discovery in the human genome, for resequencing and polymorphism discovery in *Caernohabditis elegans* [8], and for other applications such as ChIP-seq [9], which identifies genomic regions bound by transcription factors.

The very short reads—currently 30–35 bp—produced by CRT technologies such as Solexa present a far more difficult assembly problem. Standard assembly algorithms such as Arachne [10],[11] and Celera Assembler [12] cannot process such short reads at all, spurring the development of several new algorithms designed for short reads, including SSAKE [13], Velvet [14], Edena [15], and ALLPATHS [16]. These latter methods can handle Solexa data (though ALLPATHS has the additional requirement that the sequences must be paired-end reads), but they produce highly fragmented assemblies when provided with whole-genome data from a bacterial genome. The inherent problem with very short reads is that every repetitive sequence longer than the read length causes breaks in the assembly.

To demonstrate the feasibility of assembling a bacterial genome from 33 bp reads, using related genomes to assist the process, we chose *Pseudomonas aeruginosa* strain PAb1, a highly virulent strain isolated from a frostbite patient. *P. aeruginosa* is a ubiquitous environmental bacteria of clinical importance as the leading cause of gram-negative nosocomial infections [17],[18]. Several *P. aeruginosa* genomes have been sequenced previously, including two laboratory strains: PAO1 (6,264,404 bp), originally isolated from a wound, and PA14 (6,537,648 bp) isolated from a burn [19],[20]. PA14 and PAO1 are ∼99% identical across the 6.05 Mbp shared by both genomes, and their similarity to PAb1 allowed us to improve the assembly and provided a means to check its accuracy. One of our goals in sequencing PAb1 was to identify genomic differences that contribute to its altered pathogenicity.

Here we report the assembly of *P. aeruginosa* PAb1 entirely from 33 bp reads, using a novel assembly strategy that takes advantage of related genomes and homologous protein sequences. The assembly is of very high quality, comparable to or better than draft assemblies produced using earlier sequencing technologies. This study shows that a novel bacterial genome can be sequenced entirely with very short read technology, without the use of paired-end sequences (which are not available from some short-read sequencers), and assembled into a high-quality genome. Even at 40-fold coverage, the amount of sequence represents just one-quarter of a single sequencing run on a Solexa instrument, which brings the sequencing cost easily within the reach of most scientists. By making all of our assembly software free and open source, we hope to further bring down the barriers to desktop whole-genome sequencing.

### Algorithm for Assembly of Very Short Reads

We generated 8,627,900 random shotgun reads from *P. aeruginosa* PAb1 using Solexa technology. All reads were exactly 33 bp in length.

We used four distinct computational steps to assemble the genome of PAb1. For the initial step, we used the comparative assembly algorithm AMOScmp [21], which aligns all reads to a reference genome, and then builds contigs based on these alignments. The algorithm gains efficiency by avoiding the costly all-versus-all overlapping step, which is particularly difficult with very short reads due to the high incidence of false overlaps [13]. We modified AMOSCmp by tuning the MUMmer software [22], which is run within AMOScmp, to look for exact matches to the reference genome of at least 17 bp, allowing at most two mismatches in each read. We found that careful trimming of the reads based on their matches to the reference produced better assemblies than un-trimmed reads. The initial assembly used 7,500,501 reads, leaving 1,127,399 as singletons (Table 1). The PAb1 genome is closer to PA14 (99.4% identical for 92% of the PAb1 genome) than to PAO1 (99.0% identical for 90% of the PAb1 genome), and we therefore used PA14 as the primary reference for orienting the contigs.

**Table 1:** Major steps in the assembly of *P. aeruginosa* from 33 bp Solexa reads.

Assembly Step	Input	Number of Contigs	Contigs >200 bp	Largest Contig	Singletons
AMOScmp with PA14	8,627,900 reads	2,053	428	170,485	1,127,399
AMOScmp with PAO1	8,627,900 reads	2,797	865	75,626	1,592,525
Merged comparative assemblies	4,850 contigs	1,850	306	236,472	1,066,226
Gene-boosted assembly	306 contigs	120	120	512,638	NA
De novo assembly by Velvet	8,627,900 reads	10,684	7382	16,239	1,241,079
Merged gene-boosted and Velvet assemblies	120 contigs, 7382 contigs	76	76	512,638	822,210

The first column indicates the assembly strategies described in the text. Singletons refers to the number of reads that were not used to produce the contigs generated by each method.

**Figure 1:**
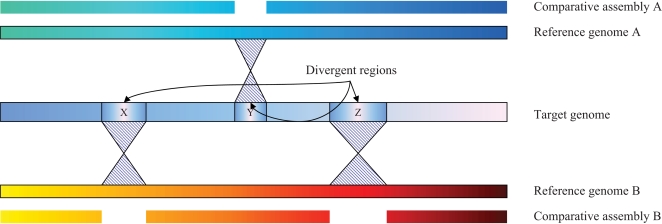
Comparative assembly using multiple genomes. The target genome is shown in the center, aligned to two related genomes, A and B. The DNA sequence of the target diverges from the reference genomes in distinct loci, labeled X, Y, and Z. The comparative assembly based on genome A contains a gap corresponding to region Y, while the assembly based on genome B contains two gaps, corresponding to X and Z. The merged assembly will cover all of the target genome with no gaps.

Our second step was a novel enhancement to the comparative assembly strategy, in which we used multiple reference genomes (Figure 1). We used the complete genomes of both PAO1 [19] and PA14 [20] separately to build multiple comparative assemblies, and found that PA14 produced the better assembly, comprising 2,053 contigs containing 6,206,284 bp. (We also used the PA7 strain, but its greater evolutionary distance made it less useful.) The bulk of the sequence was contained in 157 contigs longer than 10 Kbp, which collectively covered 5,568,616 bp. There were 331,364 bp in the PA14 genome that were not covered by the initial assembly, due to divergence between the two strains. However, the gaps in the comparative assembly based on PAO1 occurred in different locations due to differences between the strains. The best assembly based on PAO1 comprised 2797 contigs covering 6,043,652 bp.

We aligned the two assemblies to one another to identify locations where a contig in the PAO1-based assembly might span two or more contigs in the PA14-based assembly (Figure 1). For each such case, we filled the gap with the sequence from the PAO1 assembly using the Minimus assembler [23] to stitch together the contigs. This algorithm closed 203 gaps, reducing the number of contigs to 1850, of which all but 305 were <200 bp. The bulk of the genome, 5,949,162 bp, was contained in just 113 contigs of 10,000 bp or longer. Note that the overlapping contigs between the two assemblies did not agree perfectly. In order to produce a clean merged assembly, we re-mapped the reads to the contigs using AMOScmp to create consistent multi-alignments.

The third step used a novel algorithm, *gene-boosted assembly*. For this step, we took the contigs from the previous step and identified protein-coding genes using our annotation pipeline, which is based on Glimmer [24] and Blast [25]. Because amino acid sequences are much more conserved than nucleotide sequences, we were able to use the predicted protein sequences (primarily but not exclusively from other *Pseudomonas* species) to fill gaps even where the DNA sequences diverged. The annotation pipeline identified 5,769 proteins in the 305 longest contigs.

**Figure 2:**
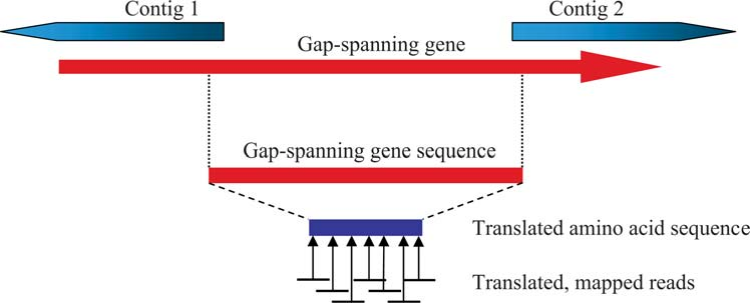
Gene-boosted assembly. All contigs are aligned with predicted gene sequences to identify genes that span 2 or more contigs. The DNA sequences of these spanning genes are cut out with a small buffer on each end. The amino acid translation of each gene fragment is then searched against a translated database of all singleton reads that have not yet been placed in the assembly. Finally, the reads identified by this process are assembled together with the two contigs to fill in the gap.

From the initial annotation, we identified those genes that extended beyond the ends of contigs or that spanned the gaps between contigs. We extracted the amino acid sequences corresponding to these gap positions, with a small buffer sequence included on each side of each gap. Next we used tblastn [25] to align each protein sequence to all the unused reads translated in all 6 frames (Figure 2). This step identified, for each gap, a small set of reads that would fill in the missing protein sequence, and the tblastn results provided initial locations for a multiple alignment. We then used a new program, ABBA (Assembly Boosted By Amino acids), to assemble the reads together with the flanking contigs and close the gaps. This gene-boosted assembly protocol extended many contigs and closed 185 gaps, ranging in length from 14–1095 bp, reducing the number of long contigs to 120.

As a separate test, we conducted a gene-boosted assembly of PAb1 using only the annotated proteins from PA14—without any reference genomic sequence. For this experiment, we aligned all the translated reads to each protein and used ABBA to assemble each one. For 4,572 of the proteins, ABBA produced a single contig that covered the entire reference protein, and another 831 proteins assembled into a few contigs. Thus 5,403 out of 5,602 (96%) of the PAb1 proteins can be assembled using a pure gene-boosting approach, and additional proteins would likely be assembled if we used a large set of proteins for boosting. This demonstrates that in the absence of a closely related genome sequence, gene-boosted assembly can use protein sequences—which diverge much more slowly than genomic DNA—to assemble most of the genes of a new bacterial strain, although the results will lack global genome structure information.

The fourth step of our method identified any remaining DNA sequences that were (a) unique to PAb1 and (b) outside predicted gene regions. We separately constructed pure *de novo* assemblies of the 8.6 million Solexa reads using SSAKE, Edena, and Velvet. The Velvet assembly was the best of the three, creating 10,684 contigs, the longest being 16,239 bp (Table 1). We used MUMmer to align these contigs to the 120 long contigs in our scaffold from the previous step, and identified cases where *de novo* contigs spanned gaps or extended contigs. This step allowed us to close 46 gaps, reducing the number of contigs in our main scaffold to 74. After removing Velvet contigs that were already contained in our scaffold, we had 436 unplaced *de novo* contigs spanning 416,897 bp. The longest unplaced contig was 10,493 bp.

## Results/Discussion

Our final assembly contains one large scaffold with 76 contigs whose total length is 6,290,005 bp, with the longest contig at 512,638 bp. The 436 unplaced contigs, which should fit into the remaining gaps, represent sequence that is unique to PAb1. Our annotation shows that most of these contigs contain genes that are homologous to other *Pseudomonas* species. Several contigs contain bacteriophage genes, pointing to recent phage insertion events in PAb1. The final assembly thus consists of 512 contigs covering 6,706,902 bp, with 94% of the bases in a single large scaffold. Approximately 9% of the reads were not used in the assembly (Table 1); many of these can be mapped to contigs if we use relaxed matching criteria, indicating that they represent low-quality data. Our annotation of the PAb1 genome identified 5,602 protein-coding genes, as compared to 5,568 for PAO1 and 5,892 for PA14.

All Solexa reads have been deposited in the Short Read Archive at NCBI, and the final genome sequence and annotation have been deposited in GenBank as sccession ABKZ01000000.

We have demonstrated that it is possible to sequence and assemble a bacterial genome from deep sequencing using 33 bp reads. The final assembly has 40.3× coverage, with very high agreement among the individual reads at the vast majority of positions in the genome. To measure the accuracy of individual reads, we examined all positions in the assembly with >20× coverage, which yielded 5.9 million positions. If we count as errors any bases that disagree with the consensus at those positions, we get an estimate based on internal consistency that the error rate per read is 1.04%. Based on this estimate, the expected number of errors for regions of the genome with coverage of >20× is close to zero, except for systematic errors such as difficult-to-sequence regions. This illustrates how the great depth of sequencing possible with short-read technology produces higher quality assemblies—in regions with deep coverage—than would conventional Sanger sequencing at a typical 8× coverage depth.

We evaluated the coverage to determine if the Solexa sequences were biased towards any portion of the genome, and found a small bias towards high-GC regions, which comprise most of the genome. In particular, regions with 60–70% GC, which comprised 79% of the genome, had 40× coverage. In contrast, regions with 50–55% GC (1.5% of the genome) had 14× coverage, and regions with <50% GC (1.1% of the genome) had just 5× coverage.

The alignment of *P. aeruginosa* PAb1 to strain PA14, which matches at 99.4% identity for >90% of the genome, can be used to provide an estimate of the sequencing accuracy. To assess the question of whether differences between our assembly and the PA14 genome represented true differences or sequencing errors, we aligned the two genomes and identified all single nucleotide polymorphisms (SNPs). Out of 5,568,550 aligned bases from the longer PAb1 contigs, 5,537,508 agreed with PA14 and can be presumed correct. For each of the remaining 31,042 SNPs, we examined all reads that were assembled at that point and assessed whether (a) the depth of coverage was adequate, and (b) the PAb1 reads agreed on the consensus base. The coverage was 10-fold or greater for 95% of these SNPs. Using the conservative assumption that a SNP might be in error if the inter-read agreement was less than 80%, we found 1157 positions (out of 5,568,550) that might be sequencing errors. We also found 1104 insertions and deletions (indels) in the aligned regions, and our assembled reads were in perfect agreement for 917 of these. If we assume conservatively that the other 187 indels are errors, then considering both SNPs and indels, the accuracy of the assembled genome is greater than 99.97%.

The assembly is sufficiently complete that we can confidently infer that genes are missing if their expected positions fall in the midst of contigs. Although deeper analysis will be presented in a followup paper, we note that the PAb1 strain is known for its hypermotility on low percentage agar media. Our sequence contains most of the genes required for swimming motility in *P. aeruginosa* [26], but is missing part of the pathway used by cyclic-di-GMP, a secondary signaling molecule, that has been implicated in repressing swimming motility [27],[28]. By searching all of the known *P. aeruginosa* genes in this pathway [29],[30],[31], we found that three genes encoding diguanylate cylase and phosphodiesterase are missing: PA2771 and PA2818 (*arr*) from the PAO1 strain, and PA14_59790 (*pvrR*) from the PA14 strain [32],[33]. All three of these genes are located in chromosomal regions previously indicated as hyper-variable based on genomic hybridizations [29]. The altered gene content of PAb1 in the regulatory pathways repressing flagella may contribute to its observed hypermotility.

The new algorithm described here make it possible for any scientist to acquire the entire genome of a bacterium at high speed and very low cost. One limitation of our method is that it depends on the existence of related genomes (for the comparative assembly step) and protein sequences (for the gene boosting step). However, GenBank already contains the complete genome sequences for >650 microbial genomes, and draft sequences for nearly 1000 more. For many of these species, much larger numbers of related strains and species have yet to be sequenced. Our method opens the door to the use of whole-genome sequencing to study entire collections of bacteria, to rapidly identify genotypes from mutagenized genetic screens, and for other analyses that were previously too costly or technically infeasible. The gene-boosted assembly technique applies equally well to both short and long-read sequencing methods, and should also work for assembling the gene-containing regions of much larger genomes.

## Methods

Genomic DNA was extracted by SDS lysis, proteinase K digest, and phenol/chloroform extraction. Sequencing was performed by Illumina using the 1G Genome Analyzer, also known as the Solexa sequencer. The 8.6 million reads represent 1/4 of the current capacity of a flow cell. For sequencing trimming in step 1, we mapped all reads to the initial assembly and then trimmed up to three bases from the 3′ end when those bases failed to match a contig. The AMOScmp pipeline for trimming and short read assembly is described at http://cbcb.umd.edu/research/SR-assembly.shtml. Contig merging in step 2 of our algorithm used the merger program from the EMBOSS package [34]. The Edena, Velvet, and ssake assemblers were run with a wide range of parameters in order to optimize them for the data used in this study, with the best results coming from Velvet with a minimum overlap requirement of 24 bases. (The other methods created more numerous, shorter contigs.) The ABBA assembler has been added to the free, open-source AMOS assembler package, which also includes the AMOScmp assembler. ABBA can be found at http://amos.sourceforge.net/docs/pipeline/abba.html. AMOS and additional modules developed in this study are freely available from http://cbcb.umd.edu/software, and the MUMmer system is freely available at http://mummer.sourceforge.net.
